# Resolving Mixed Algal Species in Hyperspectral Images

**DOI:** 10.3390/s140100001

**Published:** 2013-12-19

**Authors:** Mehrube Mehrubeoglu, Ming Y. Teng, Paul V. Zimba

**Affiliations:** 1 Hyperspectral Optical Property Instrumentation (HOPI) Laboratory, School of Engineering and Computing Sciences, Texas A&M University-Corpus Christi, 6300 Ocean Dr., Corpus Christi, TX 78412-5797, USA; 2 Center for Coastal Studies, Texas A&M University-Corpus Christi, 6300 Ocean Dr., Corpus Christi, TX 78412-*5866*, USA; E-Mail: paul.zimba@tamucc.edu

**Keywords:** hyperspectral imaging, HSI, hyperspectral imaging system, HIS, spectral response, Beer-Lambert Law, endmember extraction, linear mixing model, constrained linear unmixing

## Abstract

We investigated a lab-based hyperspectral imaging system's response from pure (single) and mixed (two) algal cultures containing known algae types and volumetric combinations to characterize the system's performance. The spectral response to volumetric changes in single and combinations of algal mixtures with known ratios were tested. Constrained linear spectral unmixing was applied to extract the algal content of the mixtures based on abundances that produced the lowest root mean square error. Percent prediction error was computed as the difference between actual percent volumetric content and abundances at minimum RMS error. Best prediction errors were computed as 0.4%, 0.4% and 6.3% for the mixed spectra from three independent experiments. The worst prediction errors were found as 5.6%, 5.4% and 13.4% for the same order of experiments. Additionally, Beer-Lambert's law was utilized to relate transmittance to different volumes of pure algal suspensions demonstrating linear logarithmic trends for optical property measurements.

## 1. Introduction

Harmful algal blooms occur frequently in both freshwater and marine systems. Evidence suggests that algal blooms have increased during the past several decades [1,2]. Algal blooms affect food webs directly by altering them when the algal toxin is produced. Indirect effects of algal blooms include changes in the quality and quantity of food resources, oxygen stress through respiring algal cells or through decomposition, and alterations of dominant algae affecting higher trophic levels. In addition, algae have been viewed as an alternative energy resource. Growing algae requires well-controlled processes where algae health is constantly monitored to adjust the algae's environmental parameters; therefore, there is a significant interest in the investigation of the properties of algae with new technologies that present themselves as fast and non-destructive solutions [3–6].

Hyperspectral imaging has been widely used in remote sensing applications [7–13]. Investigation of algal signatures using remote hyperspectral imaging has been reported by multiple research groups [12,14–18]. Craig *et al.* applied hyperspectral remote sensing for the assessment of harmful algal blooms in reflectance mode for the detection of *Karenia brevis* [16]. Szekielda *et al.* used hyperspectral imaging data collected with a portable hyperspectral imaging system in an aircraft to investigate accumulation of harmful algae, specifically cyanobacteria [17]. Oppelt *et al.* used hyperspectral imaging in remote sensing to map algal habitats using three classification techniques [18]. Casal *et al.* also reported hyperspectral remote sensing for mapping algal communities at a different location at Ria de Vigo and Ria de Aldan coast (NE Spain) [12]. Hyperspectral imaging systems in remote sensing are typically part of the payload for airborne or spaceborne systems which provide hyperspectral imagery for the end user collected in reflectance mode. For such large-scale imaging and remote sensing applications, the end-user is provided with the final imagery with preset camera and data acquisition parameters and in reflectance mode only. On the contrary, a laboratory-based hyperspectral imaging system allows experimentation under repeatable conditions. Unlike the data obtained from extraterrestrial systems, a laboratory-based system permits the adjustment of both the system and parameters for optimum data conditions for the given algal stock. The data acquisition parameters, light settings, as well as sample preparation and handling procedures can be controlled. Measurements can be taken in both reflectance and transmittance mode. Experiments can thus be conducted at a much smaller scale.

Hyperspectral imaging techniques at smaller scales have generally matured in the medical field, finding applications in skin investigations as well as in dentistry, mostly in reflectance mode [19–22]. Hyperspectral imaging has been extensively used in the agriculture and food industry [23–27]. Utility has included rapid detection of crop health issues [28,29]. In field studies, Zimba and co-authors documented algal populations in several systems with hand-held systems to assess algal communities and pond preferences of cormorants. [30,31]. In a laboratory setting, Volent *et al.* used a hyperspectral imager attached to a microscope to measure the spectral response of algae in transmittance and reflectance modes [14]. The purpose of this group's study was to separate bloom-forming algae, such as phytoplankton and macroalgae, based on the acquired spectral response that captured pigment information. No controlled mixed concentrations are reported in these papers.

We investigated the hyperspectral response of controlled mixed algal cultures containing two algal species at a time in transmittance mode to evaluate the response of a laboratory-based hyperspectral imaging system (HIS), as well as the validity of a linear spectral unmixing method in determining the composition of the mixed cultures. The ultimate goal of this project was to apply linear spectral unmixing based on linear mixing models used to predict the abundance or percent composition of algal species (endmembers) in mixed algal cultures. In this work, we compared the linear spectral unmixing results with the actual composition of the algal mixtures to assess the prediction error based on the difference between the actual concentrations and computed concentrations of algae. By using linear unmixing, linear interactions among the spectra from individual endmembers or pure algae spectra were assumed. A secondary goal of the project was to demonstrate the linear trends associated with Beer-Lambert Law and changing path length in transmission mode to allow the computation of optical properties such as the absorption coefficient using the gradient in linear logarithmic plots from hyperspectral data.

Changes in the spectral response of HIS to variations in volume and combinations of algae concentrations in transmission mode are presented. Equipment used, experimental details, data acquisition, and data conditioning are described in Section 2. The application of linear spectral unmixing to predict percent composition of algal mixtures is described in Section 3. In addition, Beer-Lambert's Law and its implementation to investigate optical properties, such as the absorptivity is presented in Section 3. Experimental results and analysis of HIS' response in differentiating algae samples and volumes are also presented in Section 4 followed by discussion and conclusions.

## 2. Instrumentation, Experiments, and Data Preprocessing

The work presented here can be separated into data acquisition, data conditioning, spectral analysis for predicting algal composition of the mixtures and for computing prediction errors, and finally application of Beer-Lambert's Law to investigate optical properties based on changing path lengths. The hyperspectral imaging system, data acquisition, noise characterization and data conditioning (preprocessing) are described in this section.

### 2.1. Hyperspectral Imaging System

**Figure 1.**
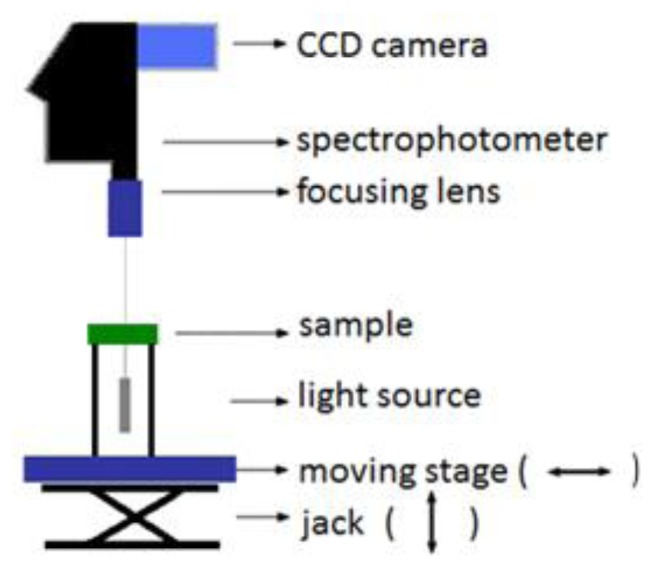
The lab-based hyperspectral imaging system in transmission mode.

Figure 1 represents the hyperspectral imaging system in transmission mode. In this configuration, the halogen broadband diffuse light source (EKE 21V 150W) illuminates the sample from the bottom. After interaction with the sample, the transmitted light is collected by the camera lens, fed into a spectrophotometer, and captured by the CDD line-scan camera, which, all together form the hyperspectral imaging system (Hyperspec™ VNIR P-Series Imaging Spectrometer, Headwall Photonics, Fitchburg, MA, USA) [32].

### 2.2. Experiments

Pure algae samples were grown at the Center for Coastal Studies laboratories in f/2 media and included the eustimatophyte *Nannochloropsis salina* (nanno), the diatom *Phaeodactylum tricornutum* (phaeo), and unidentified coccoid cyanobacteria, which represent members of the green, brown, and cyanobacterial plant line of algae. The samples varied in algae density based on growth parameters and environmental factors. The algae samples were shaken gently before hyperspectral analyses to prevent algae from settling at the bottom of the tubes or forming aggregates that could affect hyperspectral scans. Care was taken to prepare a homogenous-looking batch for experimental measurements.

Two independent set of experiments were conducted to test the hyperspectral imaging system's performance. The first set of experiments investigated spectral composition of two algal species in their pure and mixed forms. Each measurement was taken from a fixed volume of 10 mL. Spectra from pure algae (100%) and algae mixed in preset ratios (10%–90%, 50%–50%, 90%–10% combinations) were acquired and used in the constraint linear spectral unmixing model as discussed in Section 3.1 to determine the percent algae composition of the tested mixtures. Spectra from algal suspensions of 100% single-species were used as reference spectra.

The second set of experiments assessed the hyperspectral imaging system's as well as the linear spectral unmixing model's ability to differentiate among various mixed volumes of pure algae suspensions with differing concentrations of algal biomass and associated pigments related to algal growth factors. Light transmitted through 4, 6, 8 and 10 mL algae suspensions in 3.6 cm-diameter clear Petri dishes were recorded. A 2 mm-thick milky-white translucent plastic was placed under the clear Petri dishes to act as a diffuser during transmission mode measurements.

### 2.3. Data Acquisition and Conditioning

**Figure 2.**
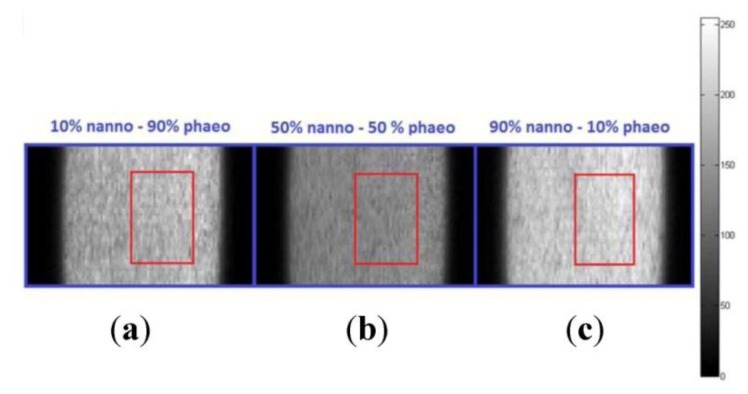
8-bit scaled image frames at 600 nm for three algae mixtures: (**a**) 10% nanno–90% phaeo; (**b**) 50% nanno–50% phaeo; (**c**) 90% nanno–10% phaeo. Red rectangles show the region of interest (frame pixels (samples and lines) to be averaged to obtain a single spectrum representing each mixture). (nanno: *Nannochloropsis salina*; phaeo: *Phaeodactylum tricornutum*).

Hyperspectral data was collected over spectral bands covering 400–1,000 nm with a spectral resolution of 3 nm. Pixel size from the camera's data sheet was 7.4 μm. Hyperspectral image cubes were acquired at spatial dimensions of 1,600 samples (fixed rows) by 50 to 150 lines (variable columns) in pixels depending on the homogeneity of the liquid samples, the step size of the horizontal moving stage, and spectral binning (number of averaged wavebands). The spectral dimension of the image was 811 bands at full spectral resolution, 406 bands with spectral binning of 2, and 271 bands with spectral binning of 3. Figure 2 shows sample image frames at 600 nm obtained from two-algae mixtures for three volumetric combinations (10%–90%, 50%–50%, 90%–10%) of pure algae. Region of interest (ROI) was selected from an area representing spatially uniform algal signal (no aggregates).

After acquisition, data preprocessing or conditioning was applied to prepare the data for linear spectral unmixing, where the computed abundances were to be compared against actual abundances to test prediction errors, thus the sensitivity of the system and validity of the data acquisition/analysis methods.

#### 2.3.1. Noise Characterization

A raw spectrum from a single pixel location across multiple frames produced a noisy signal. To characterize the noise, the expected value Ī, of the spectrum I*_i_*_,_*_j_*_,_, at location (*i*, *j*) is computed from the region of interest as in Equation (1):

(1) $$\text{E}[{\text{I}}_{i,j}]=\bar{\text{I}}=\frac{1}{MN}\sum_{i=1}^{M}\sum_{j=1}^{N}{\text{I}}_{i,j}$$

where E[I*_i_*_,_*_j_*] and Ī are the expected value of the spectrum I*_i_*_,_*_j_* obtained from a single pixel located at (*i*, *j)* across frames representing all bands, which also represent the average spectrum. [1, *M*] and [1, *N*] form the boundary of the ROI depicted in Figure 2 represented by the red rectangle with *M* = 15 and *N* = 100 pixels. The resultant average spectrum, Ī, was subtracted from a single-pixel spectrum, I*_i_*_,_*_j_*, also obtained from the region of interest to estimate the noise, N, on the spectral signal, as in Equation (2):

(2) $$\text{N}={\text{I}}_{\text{i},\text{j}}-\bar{\text{I}}$$

**Figure 3.**
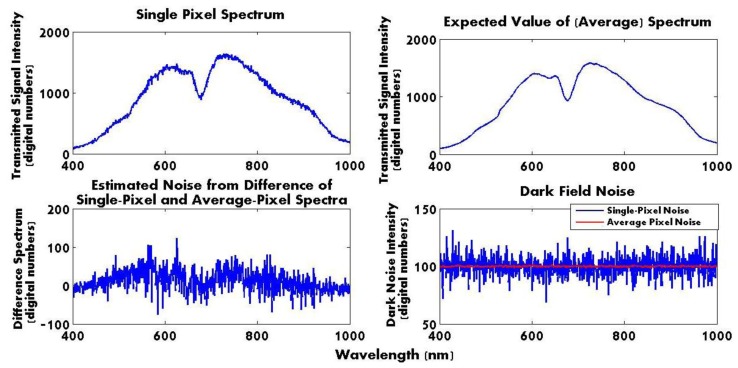
Noise characterization from a single spectrum (**Top left**), average spectrum (**Top right**), difference between the two (**Bottom left**) and dark field measurements (**Bottom right**).

N was used to compute the noise parameters, such as variance and standard deviation in the spectral dimension. Figure 3 shows the noisy single-pixel spectrum (Figure 3 Top left), the ensemble average of the spectrum within the ROI (Figure 3 Top right), and the difference between the two depicting the estimated noise (Figure 3 Bottom left). These results were compared with the dark field noise signal (Figure 3 Bottom right) from a single and average of pixels.

The noise variance in bottom left noise plot in Figure 3 was calculated to be 750.1 with a standard deviation of 27.4. The noise variance in the bottom right dark field noise plot was calculated as 71.5 with a standard deviation of 8.5. The over 10-fold difference between the variances of the dark current noise and noise estimated from the difference between a single-pixel spectrum and ensemble average of this spectrum suggests that there are other contributors to noise than just the dark field noise. The signal-to-noise ratio (SNR) was computed using Equation (3) through Equation (6):

(3) $$\text{SNR}={\left({\text{RMS}}_{\text{signal}}/{\text{RMS}}_{\text{noise}}\right)}^{2}$$

or, in decibels:

(4) SNR(dB)=10log(SNR)

where:

(5) $${\text{RMS}}_{\text{signal}}=\sqrt{\frac{1}{n}\sum_{k=1}^{n}{\bar{I}}_{\lambda_{k}}^{2}}$$

and:

(6) $${\text{RMS}}_{\text{noise}}=\sqrt{\frac{1}{n}\sum_{k=1}^{n}N_{\lambda_{k}}^{2}}$$

In Equations (5) and (6) above, *k* is the waveband (or frame number in the hyperspectral image cube), *λ_k_* is the central wavelength corresponding to the waveband *k*, and *n* is the last waveband. *N_λ_k__* is the intensity of the noise signal at wavelength *λ_k_*.

From the above equations, RMS_signal_ using the expected value of the signal spectrum was calculated as 1,005.0, and RMS_noise_ as 29.3, leading to SNR of 1,178.5 or SNR (dB) of 70.7 dB. Expected noise components include the detector and electronic noise associated with the dark field noise. Additional noise beyond the dark field noise riding on the expected value of the spectral signal is attributed to the fiber bundle used to deliver the broadband light from the source to the location of the sample contributing to additional residual spatial variations in the light field within the field of view. Such effects can be quantified by the average spatial variance. The average spatial variance across a ROI of 15 × 100 pixels at the 600 nm image frame for the spectra in Figure 3 was computed as 7,978.1 for the hyperspectral data cube used for Figure 3. The standard deviation at the same image frame (600 nm) was found as 87.9, showing the need to use an average value to smooth the data in the spatial domain and at a more selective region of interest.

Additionally, SNR was affected by the intensity or power setting of the light source. Low light source intensity settings reduced SNR as well as the spectral content of the incident light and was avoided.

#### 2.3.2. Signal Averaging and Scaling

Let us revisit the single spectrum obtained by averaging multiple spectra that corresponded to spatial locations (*i*, *j*) in the image frames within the ROI, but this time, including the band information as in Equation (7). Due to non-uniform lighting across the image frames as characterized in Section 2.3.1 above and in [32], the region of interest was selected near the center of the frame (red rectangular areas in Figure 2) and at the same loci in all algal hyperspectral images. Each pixel in the single image frame of Figure 2 is associated with a single spectral algal response represented across multiple spatial image frames at the same (*i*, *j*) location within the hyperspectral cube:

(7) $${\bar{I}}_{\lambda_{k}}=\frac{1}{MN}\overset{N}{\underset{j=1}{\text{∑}}}\overset{M}{\underset{i=1}{\text{∑}}}I_{\lambda_{k}}\left(i,j\right)$$

where *Ī_λ_k__* represents the average pixel value computed from the region of interest in a single image frame, at average wavelength, *λ*, associated with the wave band, *k*. *M* and *N* are the row and column size of the spatial 2D image frame at wave band *k*. *I_λ_k__* (*i*, *j*) is the pixel value at spatial pixel location (*i*, *j*) on the image frame associated with wavelength *λ_k_*. *k ∈* {1, 2, 3, …, *n*}. For *k* = 1, *λ_k_* = 400 nm. For *k* = *n*, *λ_k_* = 1,000 nm. When *n* = 811, no spectral binning has been applied (SB = 1). *n* = 406 corresponds to spectral binning of 2 (SB = 2), and *n* = 271 corresponds to spectral binning of 3 (SB = 3). *λ*_1_ corresponds to the 400 nm image frame. *λ_n_* corresponds to the 1,000 nm image frame.

*Ī_λ_k__* is computed for each available band, *k*, from 400 to 1,000 nm. The average single spectrum from the ROI is then represented as a vector in Equation (8), similar to Equation (1):

(8) $$\bar{\text{I}}=[{\bar{I}}_{\lambda_{1}},{\bar{I}}_{\lambda_{2}},\ldots ,{\bar{I}}_{\lambda_{k}},\ldots ,{\bar{I}}_{\lambda_{n-1}},{\bar{I}}_{\lambda_{n}}]$$

**Figure 4.**
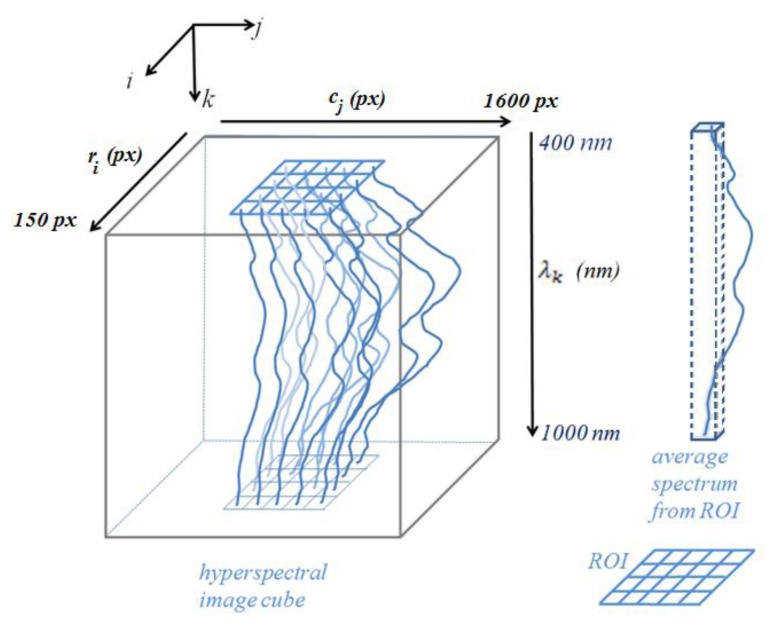
Representation of a hyperspectral image cube, and the region of interest (ROI) that is ensemble averaged to obtain a single spectrum. The ROI represents the red rectangle in Figure 2. The spectra associated with a pixel location, *(i*, *j)*, (top view) include multiple central wavelengths *λ*_1_ – *λ_n_*, where *λ*_1_ = 400 nm and *λ_n_* = 1,000 nm. *c_j_* represents the column pixel *(px)* (sample) value at location *j*; *r_i_* is the row pixel (line) value at *i*. *λ_k_* represents the different wavelengths of the spectra at wavebands *k*.

Figure 4 shows the concepts of the hyperspectral image cube, selection of region of interest, and representation of the single average spectrum from the region of interest that is represented by the red rectangle in Figure 2.

The averaging process across multiple pixels within an image frame acts as a smoothing filter reducing noise in the resultant single spectral value in that image frame. When repeated across multiple image frames at different wavebands and within the same locus of points, the result is low-noise average spectrum whose spectral properties have been minimally compromised.

In addition to the averaging process, the resultant spectrum was subsampled to only include the wavelengths 400 to 690 nm. This spectral range was selected due to the apparent discriminant spectral features showing notable variability among the observed algal mixture and endmember spectral signatures. To enhance the shape variations associated with absorption and transmittance properties within this wavelength range, the subspectrum was scaled between 0 and 1 using Equation (9) below. The results are demonstrated in Section 4:

(9) $$\bar{\text{S}}=(\bar{\text{I}}-{\bar{I}}_{\text{min}})/({\bar{I}}_{\text{max}}-{\bar{I}}_{\text{min}})$$

where S̄ is the scaled subspectral region between 400 and 690 nm for each of the observed algal sample average signatures, *Ī*_max_ is the maximum spectral signal value in Ī within this spectral region, and *Ī*_min_ is the minimum spectral value in Ī. The resultant S̄ is the scaled spectrum with intensity values between 0 and 1 (See Figure 6 in Section 4 for scaled spectral plots).

## 3. Resolving Spectral Data

### 3.1. Experiment 1—Resolving Spectra in Mixed Algal Suspensions—Linear Spectral Unmixing

Linear spectral unmixing is used for predicting the abundance (percentage) of different endmembers (pure spectra represented in each algal suspension). In this work, algal species are assumed to undergo linear interactions in the algal mixtures. The objectives of this work include the analysis and demonstration of linear separability of the two algae species and prediction of their concentration using the constrained linear mixing model. Linear spectral unmixing was achieved heuristically by computing the root mean square (RMS) error, or RMSE, for different algal combinations, and finding the abundances of the two algal species that produced the lowest RMS errors. The lowest error is assumed to occur when the correct percent combinations or abundances is identified. This value is then compared and subtracted from the known or actual combinations or abundances to compute the percent prediction error.

Hyperspectral images were acquired for 100% pure algae, and mixtures of two pure algae in volumetric combinations of 10%–90%, 50%–50% and 90%–10%, as described before. Linear spectral unmixing was implemented to determine abundances of the two algae species contributing to the overall observed spectral response in each of the mixtures [33]. Linear spectral unmixing [34–36], or endmember extraction, is a typical method used to separate spectra that belong to individual classes (endmembers) in mixed media, and to find each pure spectrum's percent contribution to the observed mixed spectrum. In this case, the model was applied to the average spectrum in the ROI for each pure or mixed algae suspension. Based on this model, abundances are related to spectral response as follows:

(10) $$\bar{\text{S}}=\sum_{i=1}^{c=2}w_{i}{\bar{\text{S}}}_{i}+\text{E}$$

where **S̄** is the observed spectrum from mixed cultures, **S̄***i* represents the spectrum from the endmember *i* (pure algal spectrum for *i*) that is assumed to contribute linearly to the observed spectrum **S̄**, *c* is the total number of algae classes or endmembers in the mixture (*c* = 2 in this case), *w_i_* represents abundance of the pure (100%) alga represented by spectrum, S̄i, and finally **E** is the error vector associated with each wavelength, *λ_k_*, as in Equation (11). *i* *∈* {1, 2} for this two-class problem:

(11) $$\text{E}=[e_{\lambda_{1}},e_{\lambda_{2}},\ldots ,e_{\lambda_{k}},\ldots ,e_{\lambda_{n-1}},e_{\lambda_{n}}].$$

For abundances, the following constraints apply:

(12) $$\sum_{i=1}^{c}w_{i}=1$$

and *w_i_* ≥ 0. From Equation (10), the error vector, **E**, is calculated as the difference between the observed and average spectrum from the mixture, and the weighted sum of pure algae average spectra.

The optimal values for abundances were found by determining the combination of abundances that produced the least error. The abundances, *w*_1_ and *w*_2_, were determined heuristically by computing the root mean square error (RMSE) for a combination of abundances where *w*_1_ was varied from 0.0000 to 100.0000% in increments of 0.0001, and *w*_2_ was varied from 100.0000 down to 0.0000% with decrements of 0.0001, conforming to the constraints of Equation (12) and non-negative *w*_2_ [33]:

(13) $$\text{RMSE}=\sqrt{\frac{1}{n}\sum_{k=1}^{n}{\left(e_{\lambda_{k}}\right)}^{2}}=\sqrt{\frac{1}{n}\sum_{k=1}^{n}{\left({\bar{I}}_{\lambda_{k}}-\left(w_{1}{\bar{I}}_{\lambda_{k}}^{1}+w_{2}{\bar{I}}_{\lambda_{k}}^{2}\right)\right)}^{2}}$$

where *Ī_λ_k__* is the average spectral value at wavelength *λ_k_* for the average raw spectrum (Equations (7) and (8)) observed from the algae mixture, ${\bar{I}}_{\lambda_{k}}^{1}$ is the average spectral pixel value at the same wavelength from pure algae class 1, and ${\bar{I}}_{\lambda_{k}}^{2}$ is the average spectral pixel value for the pure algae class 2 at *λ_k_*. Algae classes correspond to end members. *w*_1_ and *w*_2_ are the abundances of the two pure algae in the mixture.

Optimum combination of *w*_1_ and *w*_2_ coincides with the minimum error on the RMS error curve, as demonstrated in results and discussion, Section 4.1. The true concentration information of algal samples is used as the ground truth to compute prediction errors between the actual and computed concentrations using linear spectral unmixing. Percent prediction error was computed as in Equation (14):

(14) $$\text{Prediction Error}\;\left(\%\right)=\left|{\text{C}}_{\text{A}}-{\text{C}}_{\text{C}}\right|$$

where C_A_ represents the actual concentration of each alga, reported as the percentage of the mixture volume, and C_C_ represents the estimated concentration of the same alga, using the minimum RMSE value, also reported as the percentage of the mixture volume.

### 3.2. Experiment 2: Resolving Algal Volumes in Pure Algae Suspensions—Beer-Lambert Law

For the second set of experiments, pure algae samples were tested with the HIS at various volumes in transmission mode to investigate the effects on transmittance, with fixed-size Petri dishes. As summarized in [37], based on the Beer-Lambert law, the transmittance, *T*, is directly related to the path length of a sample as in Equation (15):

(15) $$T=I/I_{o}={\text{e}}^{-\text{abc}}$$

where *a* is absorptivity, *b* is path length, and *c* is concentration. *I* is the transmitted light intensity, and *I_o_* is the incident light intensity. Equation (15) leads to Equations (16) and (17) below, where:

(16) $$\log\left(I/I_{o}\right)=-\text{abc}$$

(17) $$\log\left(I\right)=-\text{abc}+\log(I_{o})$$

Provided all parameters, including concentration and wavelength, are kept constant, and only path length (volume) is changed, then relative changes in spectral response can be analyzed through transmission measurements. If the thickness of the sample is small, scattering effects can be ignored and thickness can be used to approximate the path length of the light through the medium.

Since *I_o_*, *a*, and *c* are constant across samples, Equation (17) reduces to the equation of a line, *y* = *mx* + *s*, with a constant slope, *m* = −*ac*, *y*-intercept, *s* = *log(I_o_)*, and the variable *x* = *b* or path length approximated by the sample thickness. Then *y* represents the logarithm of measured transmitted intensity, *I*. Based on Equation (17), the path length can be plotted against the logarithm of measured intensity to observe linear trends and calculate optical properties. Absorptivity, *a*, can be calculated as the slope divided by concentration, or *a* = −*m*/*c*. All parameters must be in appropriate units for the optical property interpretation. The above equation can be expanded and applied to all wavelengths in the spectrum to compute absorptivity at different wavelengths.

## 4. Results

### 4.1. Experiment 1

**Figure 5.**
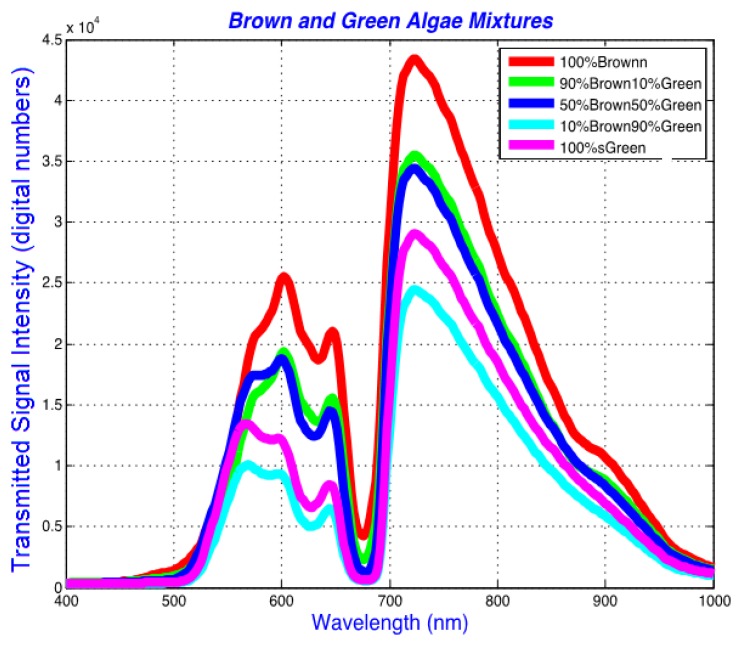
Sample average spectra showing spectral response from 100% brown algae (diatom: *Cyclotella* sp.), 100% green algae, and various volumetric mixtures between the two species. The samples represent densely grown algae, capturing absorption peaks of its key components, such as chlorophyll.

**Figure 6.**
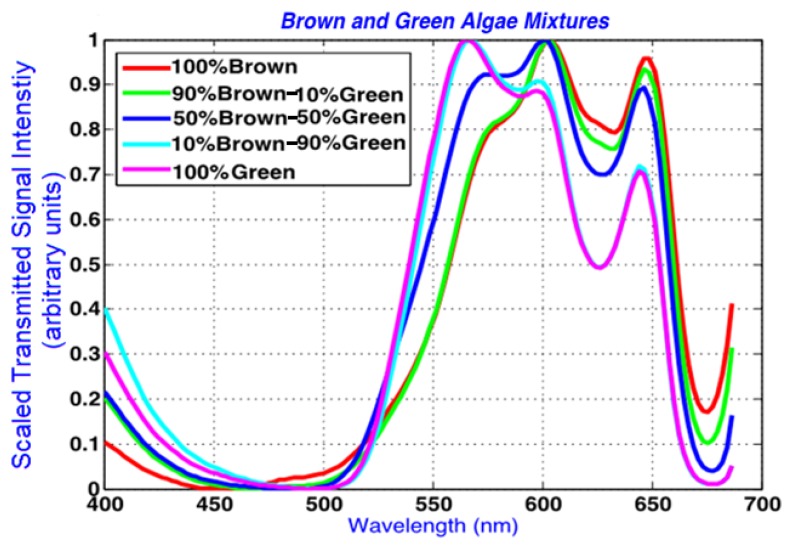
Scaled spectra from 100% green, 100% brown (diatom: *Cyclotella* sp.), and various volumetric combinations of the two. The data is scaled between 0 and 1 within 400 and 690 nm as described in Section 2.3, and demonstrates the absorption bands and peak and shoulder shifts among the algae samples more clearly.

Figures 5 and 6 show the differentiation of spectral response from two pure algae suspensions in f/2 media, and from controlled volumetric combinations (10%–90%, 50%–50%, 90%–10%) of the two. Figure 5 shows the raw spectra obtained by averaging the spectra from neighboring pixels in each image obtained by HIS. This figure represents non-normalized raw data as acquired from the system. The spectral variations among the pure and combined algae are apparent in Figure 5 particularly between about 500 and 700 nm. The absorption peak observable as the trough at around 680 nm corresponds to alpha peak of chlorophyll a (shifts to 663 nm in acetone extracted pure chlorophyll a pigment) [38], and is observed in brown, green and combined algae. The weaker absorption peak around 620 nm which appears as a shoulder or shallower valley in Figure 5 corresponds to chlorophyll a [39], and shows notable differences between brown and green algae. A smaller absorption peak for chlorophyll b occurs at around 580 nm and is cited as an absorption peak for the pure extracted chlorophyll a pigment [40]. This peak is the third highest absorption peak of chlorophyll a within 500 and 700 nm [38,39]. Although the spectral region around the 730 nm also shows spectral intensity variations among the spectra, the spectral peak around this region is not unique to either alga; therefore, the 730 nm spectral region was not included in the analysis.

In Figure 6, scaled spectra are drawn in the spectral region of interest, demonstrating the spectral shifts from pure algal spectral peaks to those in the combined algal mixtures. The data is scaled between 0 and 1 for the wavelengths between 400 and 690 nm. This wavelength range captures the main absorption peaks observed at the spectral dips in the spectra that were also identified by Volent *et al.* [14].

**Figure 7.**
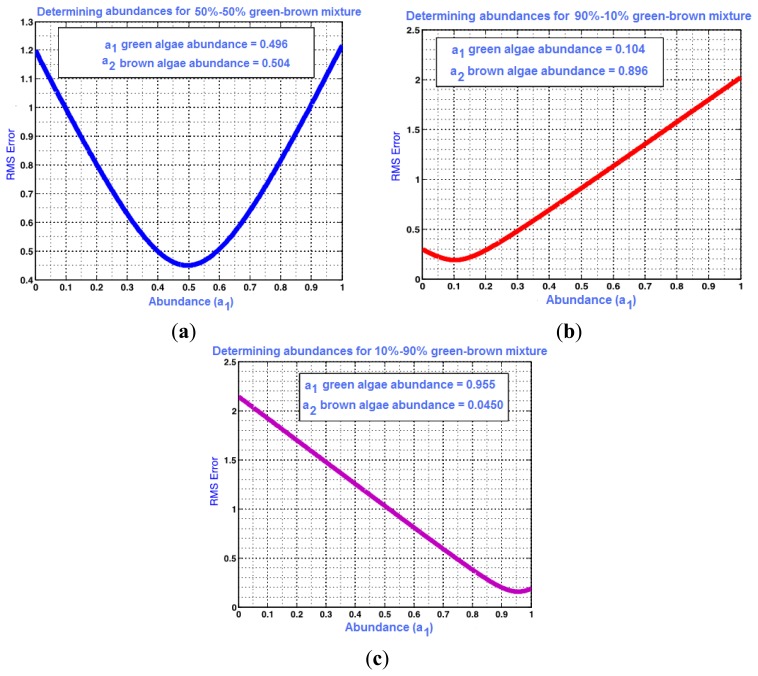
Computed abundances from algae mixture hyperspectral measurements based on the minimum error between assumed abundances and pure (single-algae) spectral combinations. The combined volumes for the green and brown algae were found to be (**a**) 10.4% and 89.6% for 10%–90% mix0ture; (**b**) 49.6% and 50.4% for the 50%–50% mix0ture; and (**c**) 95.6% and 4.5% for the 90%–10% mixture.

Using the constrained linear unmixing method described in Section 3.1, root mean square error graphs are obtained for the combined algae. The RMSE values for the above data set are plotted in Figure 7, and show the optimal abundance values that correspond to minimum error. Abundances of 10.4%–89.6%, 49.6%–50.4%, and 95.6%–4.5% were predicted for the 10%–90%, 50%–50% and 90%–10% green and brown algae combinations, respectively. The lowest prediction error was computed as 0.4% for the 10%–90% and 50%–50% green and brown algal mixtures. The prediction error was the highest in the 90%–10% green and brown algae combinations at 5.6%.

The experiments were repeated twice with independent sets of algae, each set composed of two different algae species: Comparable results were obtained with *Nannochloropsis salina* and *Phaeodactylum tricornutum* combinations (Algae Set 2). Prediction error (|actual concentration—calculated concentration|) increased with the green and blue-green algae mixtures (Algae Set 3). This third set is based on the data reported in [37]. Table 1 summarizes the results of abundance computations from all three independent experiments and data sets.

### 4.2. Experiment 2

**Figure 8.**
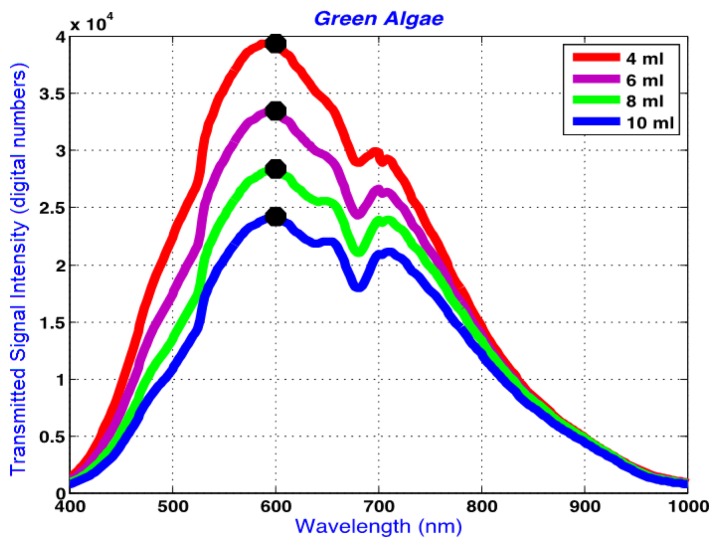
Average raw spectra from green algae samples (from set 3 in Table 1; sparsely grown) with 4, 6, 8 and 10 mL. Data points at 600 nm are selected and substituted in the trasmision equation based on Beer-Lambert Law.

Figure 8 shows the raw average spectra obtained from green algae suspensions of 4, 6, 8 and 10 mL. The horizontal axis represents the wavelengths in nm which range from 400 to 1,000 nm. This range is limited by the response of the hyperspectral camera's silicon-based imager.

The absorption peaks at around 640 and 680 nm are apparent in this figure, and correspond to absorption peaks by chlorophyll b (640 nm) and chlorophyll a (680 nm [38,39]. Figure 8 demonstrates the decreasing signal intensity with increasing optical path length associated with the increasing algae volume, which results in higher absorption and lower transmission of the incident light. The signal intensity change follows a logarithmic trend in Figure 8.

**Figure 9.**
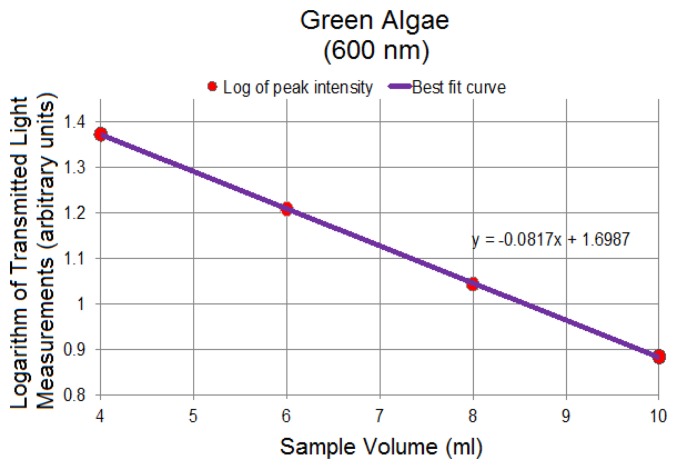
Logarithm of transmitted light intensity (reported in digital numbers) at 600 nm for 4, 6, 8 and 10 mL samples, demonstrating a linear decrease with linearly increasing algae sample volume. Gradient can be used to compute the absorptivity in respective units.

Figure 9 represents the logarithm plots for the four concentrations based on the Beer-Lambert Law applied to the data points corresponding to 600 nm in Figure 8, showing the linearity of spectral changes in the logarithmic scale at different volumes. The 600 nm was chosen arbitrarily as a representative spectral peak wavelength in transmission mode. Similar results were obtained at other selected wavelengths. The linear trends are significant in confirming the sensitivity of the system to detect small volumetric variations associated with changing path length resulting in changes in the transmitted light signal. Through this linear trend, absorptivity can be calculated based on the gradient of the plotted data and other known parameters in Equation (17).

Since all figures are based on transmitted signal strength only, and because the incident light, *I_o_*, is constant, the effect of *I_o_* on the logarithmic plots can be thought of as a DC offset, or vertical shift, of the linear graphs, preserving the integrity of the gradients. The absorptivity, an important optical property, of each solution can be calculated using the concentration information and the computed gradient. The numeric value of the gradient of the logarithmic plot in Figure 9 is −0.08. Based on the gradient, the absorptivity for the green algae of Figure 8 is −0.08/*c* where *c* is the concentration of the algal solution.

**Figure 10.**
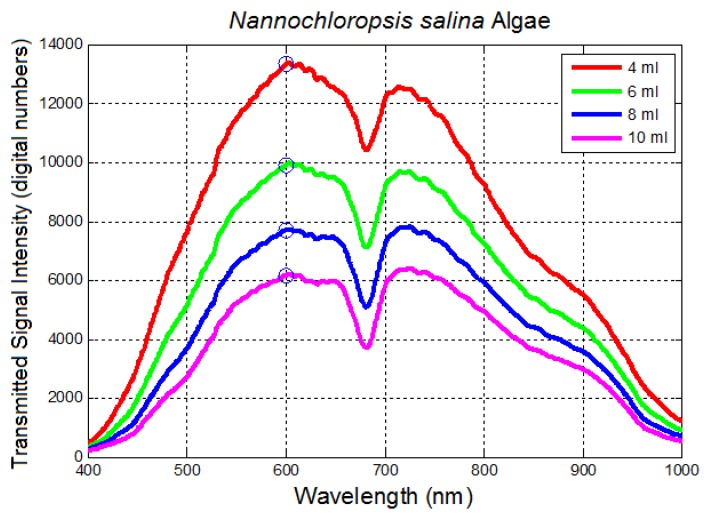
Transmitted signal intensity (average raw spectra) at different volumes of *Nannochloropsis salina* algae samples (fixed Petri dish size). 600 nm is used for logarithmic calculations, and show the expected logarithmic trends in this figure.

**Figure 11.**
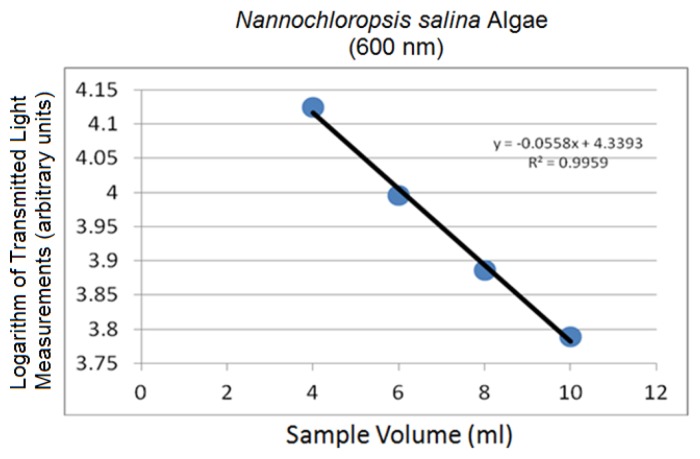
Logarithm of 600 nm spectral peak (*Nannochloropsis salina*) at different sample volumes.

**Figure 12.**
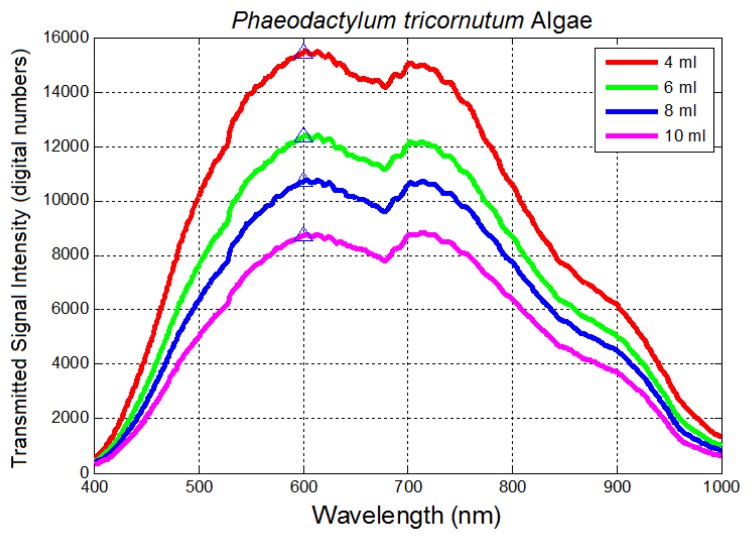
Transmitted signal intensity (average raw spectra) within 400–1,000 nm with volumes of *Phaeodactylum tricornutum* algae samples in (fixed Petri dish size); 600 nm is used for logarithmic calculations.

**Figure 13.**
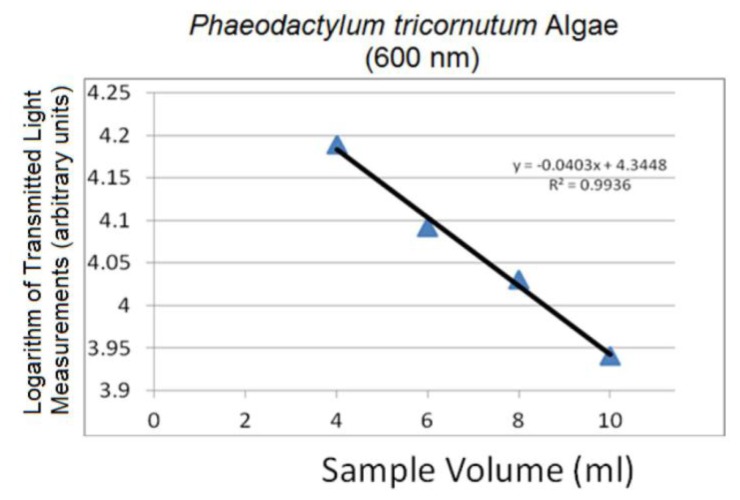
Logarithm of 600 nm spectral peak (*Phaeodactylum tricornutum*).

Linear logarithmic trends were observed in all tested algae species following Beer-Lambert's Law. Raw average spectra for *Nannochloropsis salina* and *Phaeodactylum tricornutum* algae species are plotted in Figures 10 and 12, respectively. Corresponding linear logarithmic plots are depicted in Figures 11 and 13. Figures 8, 10 and 12 depict the decreasing digital numbers recorded from the system as sample volume is increased. As can be seen in Figures 9, 11 and 13, the logarithm of raw intensity values for the algae mixtures decreases linearly as the volume of the samples increases, following Equation (17). This is because more of the incident light is absorbed by the increased volume of the liquid mixture in transmittance mode. Scattering effects are ignored in this analysis for simplicity. Scattering is less in scarcely grown algae compared to densely grown algal colonies.

The strong absorption peak of chlorophyll a at around 680 nm indicated by the dip in spectral profile at this wavelength is apparent in all Figures 8, 10 and 12. The numerical values of the gradients of the plots in Figures 11 and 13 are −0.04 and −0.06, respectively, and is the product of concentration and absorptivity after converting into appropriate units.

## 5. Discussion

During sample preparations and measurements, the algae have a tendency to settle at the bottom; therefore, it is important to acquire the hyperspectral data as quickly as possible after the samples are mixed and placed in Petri dishes. The settled algae create inhomogeneities in the suspensions, and hence affect the local spectra. Such effects are reduced by spectral averaging in samples lacking inhomogeneity such as aggregates. The region of analysis must, therefore, be carefully and consistently chosen for a given experimental data set to avoid extreme spectral fluctuations. Algae cultures must be carefully prepared and measured to ensure homogenously distributed batches and samples in Petri dishes.

In the case of mixed algae, green and blue-green algae results produced the highest prediction errors compared to the other two algae sets. Several factors contributed to these results. First, the spectral signatures of the two algae were very similar. Second, the algae samples were the most sparsely grown among the three sets. Although care was taken to avoid algae binding together, dark spots associated with chunks of algae were observed in the measured mixtures and images, introducing error. In addition, this data set was taken with spectral binning of 3, further smoothing the spectral response and fine details of the spectra during acquisition. The error range for this set was between 6.3% and 13.4% compared to other two sets whose error range was between 0.4% and 5.6%, as reported in Table 1. So the spectral binning during acquisition is expected to have also removed subtle differences in the spectra of these already-sparse algal suspensions.

The Beer-Lambert Law was applied and path length was assumed to be exchangeable with sample thickness as volume of the samples was increased in a fixed-size Petri dish. The sample holders, or Petri dishes, were 3.6 cm in diameter, or of 1.8 cm radius, *r*. The volume, *V*, of the Petri dishes was calculated as the volume of a cylinder. With a fixed area, *A* = Π*r*^2^ or 10.18 cm^2^, the height, *h*, depends on the sample volume added. For each milliliter of algae, the volume changes by 1 cubic centimeter. Therefore, since *h* = *V*/*A*, for every 2 mL algae sample added to the Petri dish, the height, *h*, increased by 1.96 mm (*h* = 2/(Π(1.8)^2^) = 0.196 cm), translating to approximately 2 mm per 2 mL of sample as plotted in Figures 9, 11 and 13. Considering the low density of algae in the media solution, sample height in the Petri dishes were approximated for path length, and linear results were obtained as expected for linear increases in volume.

It is noted that although linear interactions are assumed among the mixed algal cultures, nonlinearities are expected due to the size distribution and translucent liquid nature of the growth medium with algae. For example, scattering from neighboring regions that appear to come from the observed pixel but does not belong to the observed pixel spectrum [41] is ignored. We expect that the linearity assumption also adds to the prediction error, and must be investigated further.

## 6. Conclusions

Two sets of experiments were conducted to characterize a lab-based hyperspectral imaging system's capability to resolve, first, individual algae species in mixed algae suspensions containing two algae species, and, second, volumetric changes from pure algal batches.

Using a linear spectral unmixing algorithm for reflectance data, abundances were computed with prediction errors between 0.4% and 5.6% for two data sets and between 6.3% and 13.4% for one data set. Raw hyperspectral data from dense algal growth produced stronger algae signatures, whereas sparsely grown algae produced signatures that were dominated by the growth medium.

Small volumetric changes were successfully captured in transmission mode using the hyperspectral imaging system for both densely and sparsely grown pure algae suspensions. Although many factors affect the data quality, in general, more accurate results were obtained from data acquired with no spectral binning or spectral binning of two, as well as homogenously and densely grown species of algal suspensions.

More complex algorithms can be used to assist with solving the reverse problem in media characterization and algal quality monitoring, once baseline information for optimum conditions are established with the tested technology. In particular, non-linear models that capture non-linear interactions between the algae species and growth medium should be investigated to reduce prediction errors.

## Figures and Tables

**Table 1.** Actual and computed algae concentrations via linear spectral unmixing (LSU) and minimum RMS error. A variety of algal species were tested in three experiments under different settings for spectral binning (SB). (Nanno: *Nannochloropsis salina*; Phaeo: *Phaeodactylum tricornutum*; Green: coccoid cyanobacteria of green and brown plant lines (two separate green algae used in Experiments 1 and 3); Brown: diatom (Cyclotella sp.)) Concentrations are listed by mixed volume.

**Experiment (Algae Set)**	**Algae Mixtures**	**Actual Concentration (%) (Ground Truth)**	**Computed Concentration (%) (LSU)**	**Prediction Error (%) (|Computed−Actual|)**
**1**	**Green (G) and Brown (Diatom: Cyclotella sp.) (B) (SB = 2)**	10–90	10.4–89.6	0.4
		G–B	G–B	
		50–50	49.6–50.4	0.4
		G–B	G–B	
		90–10	95.6–4.5	5.6
		G–B	G–B	

**2**	*Nannochloropsis salina* (N) and *Phaeodactylum tricornutum* (P) **(SB** = **1)**	10–90	8.3–91.7	1.7
		N–P	N–P	
		50–50	50.4–49.6	0.4
		N–P	N–P	
		90–10	95.4–4.6	5.4
		N–P	N–P	

**3**	Green (G2) and Blue-Green (BG) **(SB **= **3)**	10–90	16.6–83.4	6.6
		G2–BG	G2–BG	
		50–50	36.6–63.4	13.4
		G2–BG	G2–BG	
		90–10	83.7–16.3	6.3
		G2–BG	G2–BG	

## References

[1] Hallegraeff G.M. (1993). A review of harmful algal blooms and their apparent global increase. Phycologia.

[2] Shumway S.E. (1990). A review of the effects of algal blooms on shellfish and aquaculture. J. World Aquac. Soc..

[3] Gao Y., Yu Y., Liang J., Gao Y., Luo Q. Isolation of Four Diatom Strains from Tidal Mud toward Biofuel Production.

[4] Taulant M., Chen M., Holland T., Basu A. Optical Microplates for Photonic High Throughput Screening of Algal Photosynthesis and Biofuel Production.

[5] Renita A.A., Amarnath D.J., Padhmanabhan A., Dhamodaran B., Kizhakudan J. Production of Bio-Diesel from Marine Macro Algae.

[6] Pienkos P.T., Jarvis E., Darzins A. (2010). Green Gold. IEEE Spectr..

[7] Al-Moustafa T., Armitage R.P., Danson F.M. (2012). Mapping fuel moisture content in upland vegetation using airborne hyperspectral imagery. Remote Sens. Environ..

[8] Themelis K.E., Schmidt F., Sykioti O., Rontogiannis A.A., Koutroumbas K.D., Daglis I.A. (2012). On the unmixing of MEx/OMEGA hyperspectral data. Planet. Space Sci..

[9] Féret J.-B., Asner G.P. (2012). Semi-supervised methods to identify individual crowns of lowland tropical canopy species using imaging spectroscopy and LiDAR. Remote Sens..

[10] Robichaud P.R., Lewis S.A., Laes D.Y.M., Hudak A.T., Kokaly R.F., Zamudio J.A. (2007). Postfire soil burn severity mapping with hyperspectral image unmixing. Remote Sens. Environ..

[11] Asner G.P., Heidebrecht K.B. (2002). Spectral unmixing of vegetation, soil and dry carbon cover in arid regions: Comparing multispectral and hyperspectral observations. Int. J. Remote Sens..

[12] Casal G., Sánchez-Carnero N., Domínguez-Gómez J.A., Kutser T., Freire J. (2012). Assessment of AHS (Airborne Hyperspectral Scanner) sensor to map macroalgal communities on the Ria de vigo and Ria de Aldan coast (NW Spain). Mar. Biol..

[13] Liao W., Bellens R., Pižurica A., Philips W., Pi Y. (2012). Classification of hyperspectral data over urban areas using directional morphological profiles and semi-supervised feature extraction. IEEE J. Sel. Top. Appl. Earth Obs. Remote Sens..

[14] Volent Z., Johnsen G., Sigernes F. (2009). Microscopic hyperspectral imaging used as a bio-optical taxonomic tool for micro- and macroalgae. Appl. Opt..

[15] Lekki J., Anderson R., Nguyen Q.-V., Demers J., Leshkevich G., Flatico J., Kojima J. Development of Hyperspectral Remote Sensing Capability for the Early Detection and Monitoring of Harmful Algal Blooms (HABs) in the Great Lakes.

[16] Craig S.E., Lohrenz S.E., Lee Z., Mahoney K.L., Kirkpatrick G.J., Schofield O.M., Steward R.G. (2006). Use of hyperspectral remote sensing reflectance for detection and assessment of the harmful alga. Karenia brevis. Appl. Opt..

[17] Szekielda K.H., Marmorino G.O., Maness S.J., Donato T.F., Bowles J.H., Miller W.D., Rhea W.J. (2007). Airborne hyperspectral imaging of cyanobacteria accumulations in the Potomac River. J. Appl. Remote Sens..

[18] Oppelt N.M., Schulze F., Doernhoefer K., Eisenhardt I., Bartsch I. (2012). Hyperspectral classification approaches for intertidal macroalgae habitat mapping: A case study in Heligoland. Opt. Eng..

[19] Zuzak K.J., Perumanoor T.J., Naik S.C., Mandhale M., Livingston E.H. A Multimodal Reflectance Hyperspectral Imaging System for Monitoring Wound Healing in Below Knee Amputations.

[20] Li Q., Liu J., Xiao G., Xue Y. Hyperspectral Tongue Imaging System used in Tongue Diagnosis.

[21] Roblyer D.M., Kurachi C., El-Naggar A., Williams M.D., Gillenwater A., Richards-Kortum R. Multispectral and Hyperspectral *in vivo* Imaging of the Oral Cavity for Neoplastic Tissue Detection.

[22] Usenik P., Bürmen M., Fidler A., Pernuš F., Likar B. (2012). Automated classification and visualization of healthy and diseased hard dental tissues by near-infrared hyperspectral imaging. Appl. Spectrosc..

[23] Li Y., Shan J., Peng Y., Gao X. Nondestructive Assessment of Beef-Marbling Grade Using Hyperspectral Imaging Technology.

[24] Zhou Y., Mao H., Zhang X. Hyperspectral Imaging Technology for Detection of Moisture Content of Tomato Leaves.

[25] Ma B., Xiao W., Qu N., Wang W., Wang L., Wu J. Detection of Fruits Slight Bruises Based on Hyperspectral Imaging Technology.

[26] Zhao J., Chen Q., Cai J., Ouyang Q. (2009). Automated tea quality classification by hyperspectral imaging. Appl. Opt..

[27] Li J., Xue L., Lui M., Wang X., Luo C. (2010). Hyperspectral imaging technology for determination of dichlorvos residue on the surface naval orange. Chin. Opt. Lett..

[28] Zimba P.V., Gitelson A.A. (2006). Remote estimation of chlorophyll concentration in hyper-eutrophic aquatic systems: Model tuning and accuracy optimization. Aquaculture.

[29] Grisham M.P., Johnson R.M., Zimba P.V. (2010). Detecting Sugarcane yellow leaf virus infection in asymptomatic leaves with hyperspectral remote sensing and associated leaf pigment changes. J. Virol. Methods.

[30] Radomski A., Zimba P.V. (2010). Does pond water reflectance influence double-crested Cormorant selection of aquaculture ponds?. J. World Aquac. Soc..

[31] Ritchie J.E., Zimba P.V., Everrit J. (2003). A review of ARS sponsored remote sensing research in aquatic systems. Photogrammic Eng. Remote Sens..

[32] Mehrubeoglu M. Spectral Characterization of a Hyperspectral Imaging System Using Optical Standards.

[33] Sabol D.E., Gillespie A.R., Adams J.B., Smith M.O., Tuckerb C.J. (2002). Structural stage in Pacific Northwest forests estimated using simple mixing models of multispectral images. Remote Sens. Environ..

[34] Bin L., Chenghai Y., Chanussot J. Linear Unmixing of Multidate Hyperspectral Imagery for Crop Yield Estimation.

[35] Zhang D., Yin J., Li H. Mapping of Surface Sediment Types in Intertidal Flat Using Linear Spectral Unmixing of Hyperion Data.

[36] Silván-Cárdenas J.L., Wag L. (2010). Fully constrained linear spectral unmixing: Analytic solution using fuzzy sets. IEEE Trans. Geosci. Remote Sens..

[37] Mehrubeoglu M., Teng M.Y., Savage M., Rafalski A., Zimba P.V. Hyperspectral imaging and analysis of mixed algae species in liquid media.

[38] Weaver E., Wrigley R. (1994). Factors affecting the identification of phytoplankton groups by means of remote sensing. NASA Tech. Memo..

[39] Absorption Spectra of Chlorophyll a (Light Green) and Chlorophyll b (Turquoise). http://www.biologie.uni-hamburg.de/b-online/e24/3.htm.

[40] Chappelle E.W., Kim M.S., McMurtrey J.E. (1992). Ratio analysis of reflectance spectra (RARS): An algorithm for the remote estimation of the concentrations of chlorophyll A, chlorophyll B, and carotenoids in soybean leaves. Remote Sens. Environ..

[41] Bioucas-Dias J.M., Plaza A., Dobigeon N., Parente M., Du Q., Gader P., Chanussot J. (2012). Hyperspectral unmixing overview: Geometrical, statistical, and sparse regression-based approaches. IEEE J. Sel. Top. Appl. Earth Obs. Remote Sens..

